# Magnesium and Zinc Oxide Nanoparticles from *Datura alba* Improve Cognitive Impairment and Blood Brain Barrier Leakage

**DOI:** 10.3390/molecules27154753

**Published:** 2022-07-25

**Authors:** Habib Ullah, Ikram Ullah, Gauhar Rehman, Muhammad Hamayun, Sajid Ali, Abdur Rahman, In-Jung Lee

**Affiliations:** 1 Department of Zoology, Abdul Wali Khan University, Mardan 23200, Pakistan; zoologist399@gmail.com (H.U.); 03149443357ar@gmail.com (A.R.); 2 Sulaiman Bin Abdullah Aba Al-Khail-Centre for Interdisciplinary Research in Basic Sciences, Faculty of Basic and Applied Sciences, International Islamic University, H-10, Islamabad 44000, Pakistan; 3 Department of Botany, Abdul Wali Khan University, Mardan 23200, Pakistan; hamayun@awkum.edu.pk; 4 Department of Horticulture and Life Science, Yeungnam University, Gyeongsan-si 38541, Korea; drsajid@yu.ac.kr; 5 Department of Applied Biosciences, School of Life Sciences Kyungpook National University, Daegu 41566, Korea

**Keywords:** magnesium oxide, nanoparticles, pentylenetetrazole, *Datura alba*, blood brain barrier, seizures

## Abstract

Epilepsy is a neurological disorder involving persistent spontaneous seizures and uncontrolled neuronal excitability that leads to cognitive impairments and blood–brain barrier (BBB) disruption. Currently available antiepileptic drugs present side effects and researchers are trying to discover new agents with properties to overcome these drawbacks. The aim was to synthesize magnesium oxide (MgO) and zinc oxide (ZnO) nanoparticles from *Datura alba* fresh leaf extracts and evaluate their anti-epileptic potential in mice kindling or a repetitive seizures model. The phytoassisted synthesized nanoparticles were characterized using spectroscopy; FT-IR, XRD, SEM, and EDX. Analysis of the NPs confirmed the crystalline pleomorphic shape using the salts of both zinc and magnesium possibly stabilized, functionalized and reduced by bioactive molecules present in plant extract. By using several characterization techniques, NPs were confirmed. UV-Vis spectroscopy of biologically produced ZnO and MgO revealed distinctive peaks at 380 nm and 242 nm, respectively. Our findings categorically demonstrated the reductive role of biomolecules in the formation of ZnO and MgO NPs. The mice kindling model was induced using seven injections of Pentylenetetrazole (PTZ, 40 mg/kg, i.p) for 15 days alternatively. The results showed that mice post-treated with either ZnO or MgO nanoparticles (10 mg/kg, i.p) significantly improved in respect of behavior and memory as confirmed in the Morris water maze (MWM), open field (OF), novel object recognition (NOR) test compared with PTZ treated mice. Furthermore, the ZnO and MgO nanoparticle treatment also maintained the integrity of the BBB, reducing the leakage, as confirmed by Evans blue dye (EBD) compared with PTZ treated mice only. In summary, the current finding demonstrates that green synthesized ZnO and MgO nanoparticles have neuroprotective, ant-epileptic potential, molecular mechanisms, and clinical implications need to be further explored.

## 1. Introduction

Epilepsy is a chronic central nervous system disorder causing recurrent seizures. The abnormal neuronal activity causes such condition [1]. Various cognitive, psychological, neurobiological signs and symptoms are associated with epilepsy. Developing countries present a relatively higher prevalence than developed countries. The World Health Organization (WHO) reported in 2018 that globally it is fourth most occurring neurological disorder after stroke, migraine, and Alzheimer’s disease. There are more than 30 different types of epileptic syndromes with more than 15 types of seizures that affects approximately 65 million people globally [2]. Neuronal excitation is controlled by several different neuromodulators and neurotransmitters. However, L-glutamate and gamma-amino butyric acid (GABA) are two important excitatory and inhibitory neurotransmitters respectively in the mammalian brain. Alteration in the function of these two systems may be associated with epilepsy managed by antiepileptic drugs (AEDs), which modulate neurotransmitters or ion channels that eventually affects neuronal excitability in brain. Despite current advances, approximately 30–40% of epileptic patients experience refractory seizures with other side effects after the administration of AEDs [3]. Ischemia, depression, anxiety, motor disability, mode alterations, cognitive deficit, and tremor are common side effects [4]. A majority of AEDs mitigate severity of seizure but do not prevent the progression of disease. Owing to the side effects and the resistance of patients to synthetic AEDs, new strategies are being focused to develop novel therapeutics to modify disease progression along with suppression of seizure [5].

For screening of anticonvulsant agents, chemical Kindling is widely used experimental model. Kindling is produced by repeated injections of sub-convulsive dose of pro-convulsant drug resulting in seizure. Pentylenetetrazole (PTZ) is non-competitive GABA-A receptor chloride channel antagonist and used to develop chemical induced kindling in rats and mice at sub-convulsant doses [4]. Furthermore, PTZ causes glutamatergic system dysfunctions, induce anxiety and depression. In addition, the repetitive administration of PTZ causes neuronal loss in the hippocampus, as well as alterations in the emotional behavior and cognitive inconsistencies. PTZ can be used for developing chronic as well as acute animal models of epilepsy. For example, repetitive administration at sub-threshold doses (20–40 mg/kg, i.p) produces kindling while acute models can be produced by a single threshold dose (60–100 mg/kg, i.p) [6].

Green Nanotechnology is an emerging field dealing with nanostructure having dimension of 1–1000 nanometer. Green synthesis is a safe, one step, ecofriendly and cost-effective method for generation of biocompatible metal oxide. NPs biosynthesis involves the usage of biomaterials (e.g., plant extracts, algae, bacteria, and fungi). Phytosynthesis is considered a promising route among biosynthesis methods and literature have reported that leaf extract mediated NPs have better bioactivity [7]. Recently, NPs based therapy of brain diseases and disorders have attracted attention due to its transportability across BBB, less toxicity, solubility and biodegradability [8].

*Datura alba* (Devil’s Trumpet) is a perennial plant belonging to family Solanaceae having average height of 4.9 ft. *Datura* is a common medicinal plant used to cure ailments like asthma, cough, convulsions, and insanity. The seeds and leaves are often used as bronchodilators, antispasmodics, anesthetics, and hallucinogens in herbal medicine. As a bronchodilator, anesthetic, anodyne, anti-asthmatic, antispasmodic, anti-tussive, and hallucinogenic, the leaves and seeds in particular are employed. The plant is effective in treating catarrh, diarrhea, and skin disorders. Rheumatic pains, hemorrhoids, painful menstruation, catarrh, diarrhea, epilepsy, insanity, hysteria, skin ulcers, and wounds are among the conditions it is used to treat. Various isolated compounds of *D. alba* are currently used against different neurological disorders, including epilepsy and insanity [9]. Phytochemical analysis of leaf shows copious number of flavonoids, phenols, tannins, and alkaloids [10]. This prompted us to use biometabolites of *D. alba* leaves extracts as stabilizing and reducing agents for biosynthesis of ZnO and MgO NPs.

MgO and ZnO NPs have wide variety of biomedical applications against various diseases including neurological disorders as revealed by different studies. ZnO NPs have anticancer, antidiabetic, antibacterial and antioxidant activity. Similarly, MgO NPs are used as anti-inflammatory, anti-fungal, antibacterial, and anxiolytic agents [11]. However, to the best of our knowledge, there is no report available regarding the role of biosynthesized MgO and ZnO NPs in relation to memory improvement in a PTZ induced epileptic mice model. In addition, impact of these NPs on neurobehavior in other animal models been studied. Zinc is a biologically essential neuromodulator metal found in high level in synaptic vesicles of glutamatergic neuron population throughout hippocampus, cerebral cortex, auditory brainstem and striatum. The hippocampus is actively involved in memory and learning processes. *N*-methyl-d-aspartate (NMDA) is involved in epilepsy and depression. Co-release of Zinc with glutamate in presynaptic space inhibits NMDA receptors activation via different mechanisms. In the hippocampus, it decreases glutamate release from presynaptic neuron by enhancing synthesis and exocytosis of GABA [12]. MgO NPs enhance locomotor activity and reduce anxiety i.e., behavior in morphine induced mice model [13]. Magnesium is required for the normal functioning of different organ systems, including the CNS. Being the fourth most abundant metal in body, it is a co-factor of more than 300 enzymes. Several studies have reported that Mg^2+^ critically modulates NMDA, GABA_A_, and voltage gated channels. It plays a critical role in neuronal plasticity, memory, and learning. Its supplementation mitigates anxiety and improves cognitive impairment in neurodegenerative diseases [14].

Considering previous literature, we aimed to synthesize ZnO and MgO NPs using *Datura alba* leaves extracts and investigate their possible role in learning, memory, and anxiety in a PTZ-induced epileptic mice model. The biogenic synthesis of these NPs using the extracts of this plant and their role in cognition in epilepsy are considered for the first time.

## 2. Results

### 2.1. UV Visible Spectroscopy

**Figure 1:**
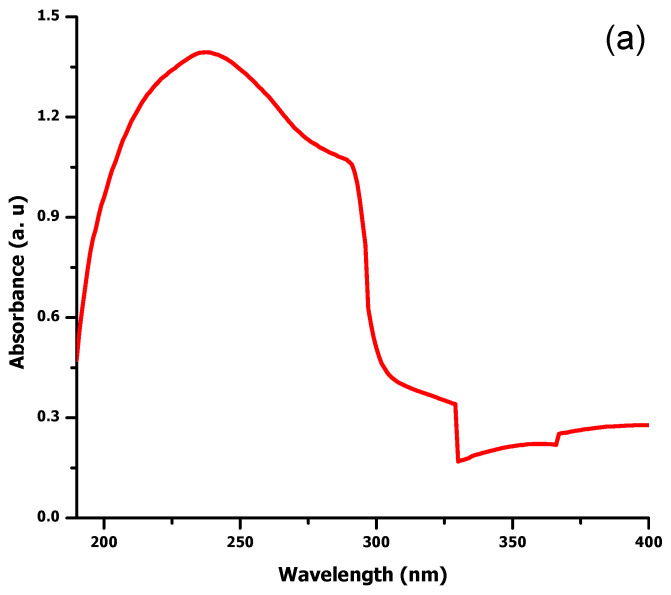

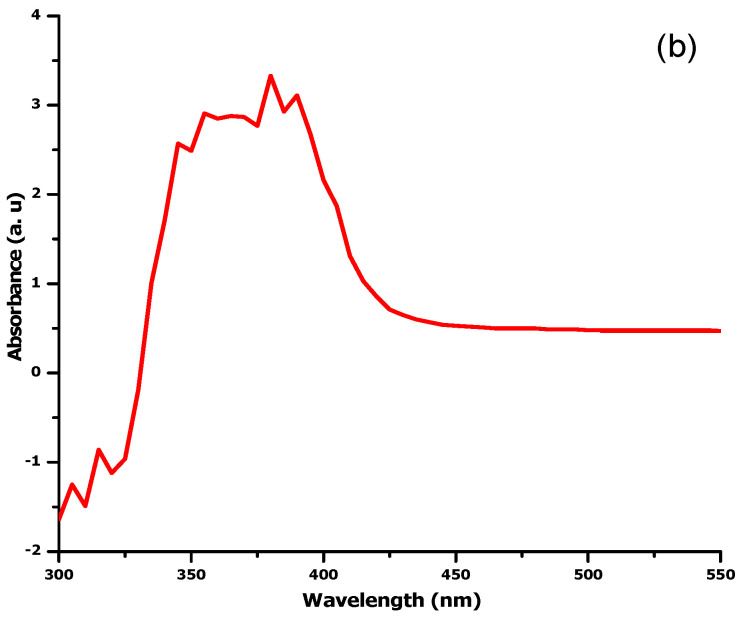
Absorption spectra of Phytosynthesized Nanoparticles using *Datura alba* leaf extracts. Panel (**a**) depicts MgO UV-vis spectra, whereas panel (**b**) depicts ZnO spectra.

Plant assisted MgO and ZnO NPs were measured by UV-vis absorption spectroscopy. Characteristic absorbance peaks of MgO and ZnO NPs were obtained at 242 nm and 380 nm respectively (Figure 1a,b). This result reveals the bio-reductive role of *D. alba* leaf extract biomolecules, leading to the successful formation of NPs.

### 2.2. Functional Group Analysis by FTIR Spectroscopy

**Figure 2:**
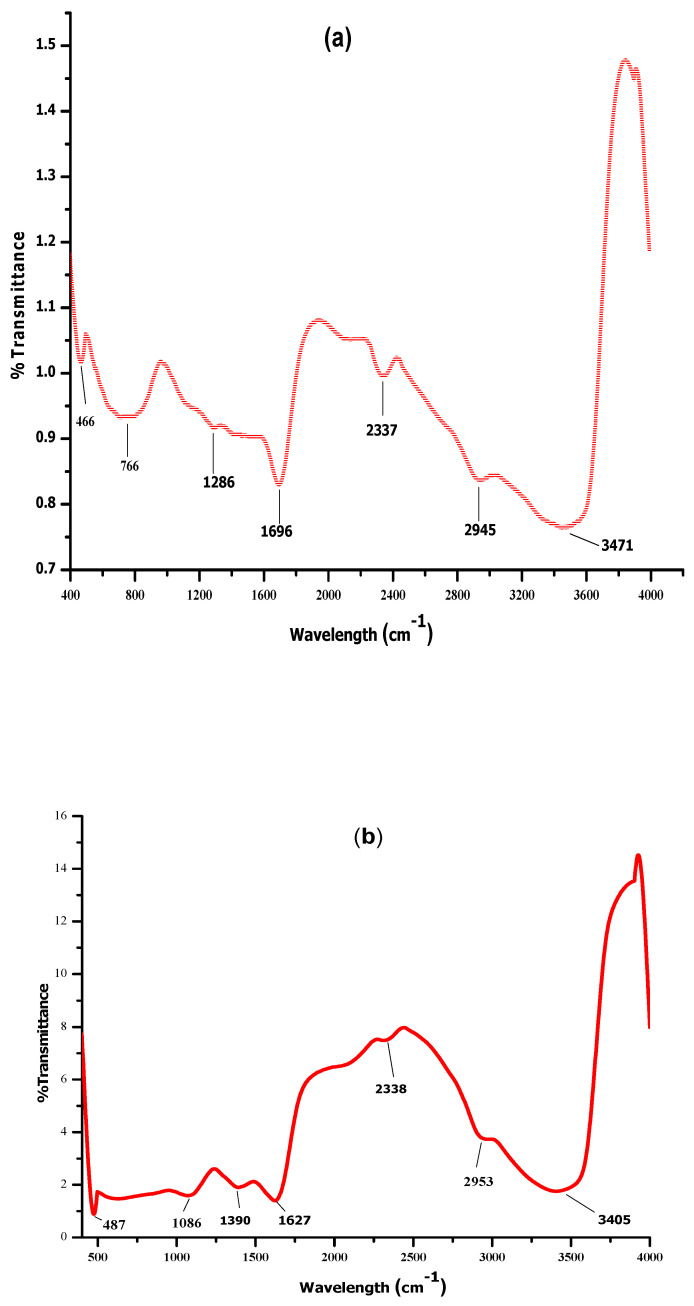
FTIR spectra of Biosynthesized Nanoparticles showing bonds of different capping biomolecules of *Datura alba*. (**a**) MgO (**b**) ZnO.

FTIR was used for the identification of biomolecules responsible for the biogenic reduction and stabilization of ZnO and MgO NPs. Figure 2a depicts peaks at 466, 766, 1286, 1696, 2337, 2945, and 3471 cm^−1^ in the FTIR spectra of *D. alba* leaf extract mediated MgO NPs. The peak at 3471 cm^−1^ shows a vibration of O-H, indicating alcohol, ether, carboxylic acid, and ester, while the 2945 cm^−1^ band is due to the vibration of the C-H group. The band at 2337 cm^−1^ is ascribed to C-H stretching in the aldehyde group, while C = C and C = O bonds were revealed by a peak at 1696 cm^−1^ represents the presence of alkenes, aldehyde, flavonoids, and ketones. Presence of alkaloids and amides were determined by 1286 cm^−1^ band. The peaks at 466 cm^−1^ and 766 cm^−1^ confirm the bond between Oxygen and Magnesium.

Figure 2b presents FTIR spectra of ZnO NPs showing a sharp peak of metal-oxygen bond at 487 cm^−1^. The bands at 1086 cm^−1^ and 1390 cm^−1^ are due to the stretching mode of C-O and C = C in amino acids and polyphenols/aromatic ring conjugates. The peaks at 2338 cm^−1^, 1630 cm^−1^, and 2953 cm^−1^ correspond to C = C and C-H stretching vibration in the amide group of protein and C-H stretching in phospholipids and alkane respectively. The broad peak at 3405 cm^−1^ signifies O-H and N-H groups in alcohol, flavonoids, and amines, respectively. FTIR examination confirmed the biosynthesis of NPs and the presence of different functional groups of *D. alba*.

### 2.3. X-ray Diffraction Analysis

**Figure 3:**
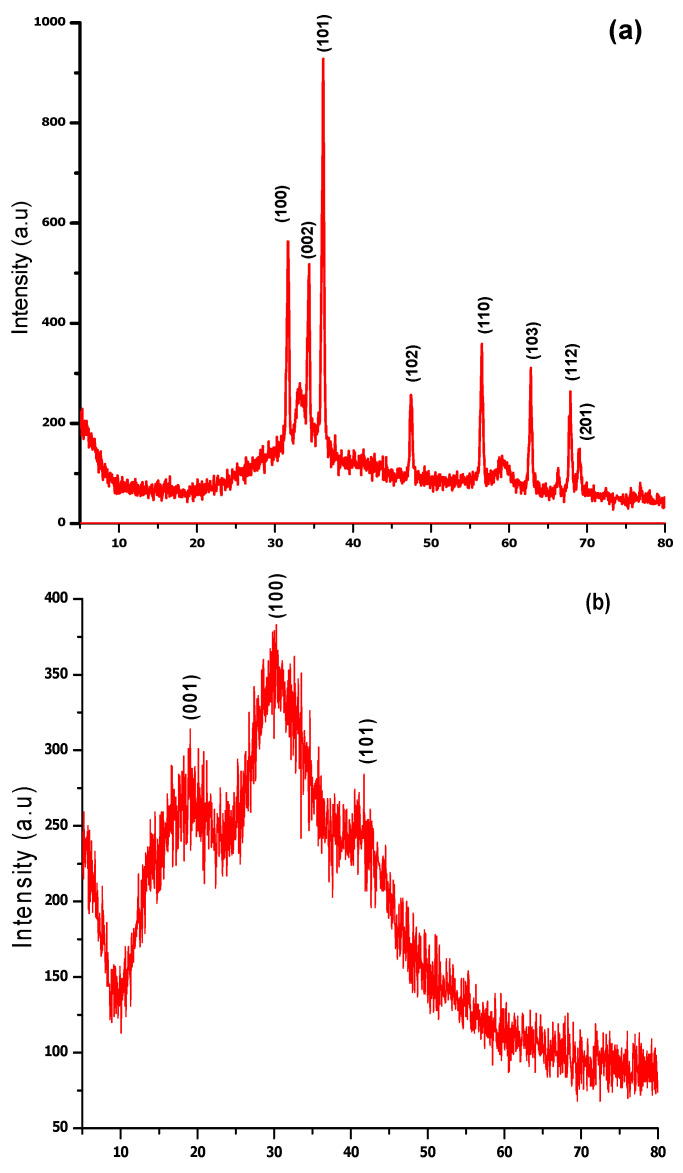
XRD pattern of (**a**) ZnO and (**b**) MgO Nanoparticles synthesized by bioreduction activity of *Datura alba* leaves extract.

Figure 3a depicts the Bragg’s reflections peaks at 2θ of 69.13°, 67.84°, 62.87°, 56.60°, 47.66°, 36.23°, 34.50°, and 31.68° corresponding to the lattice plane (201), (112), (103), (110), (102), (101), (002), and (100), respectively, found in line with Wurtzite ZnO (JCPDS file no: 043-0002). Clear and distinct peaks confirmed the high purity and crystalline nature of phytosynthesized ZnO NPs.

Figure 3b represents the diffractogram of phyto-assisted MgO NPs at 2θ = 40°, 31° and 19.14° indexed to (101), (100) and (001) respectively. The planes show good agreement with (JCPDS no: 002-1207), which confirms the crystalline structure with amorphous phase due to water and impurities.

### 2.4. SEM and EDX Analysis

**Figure 4:**
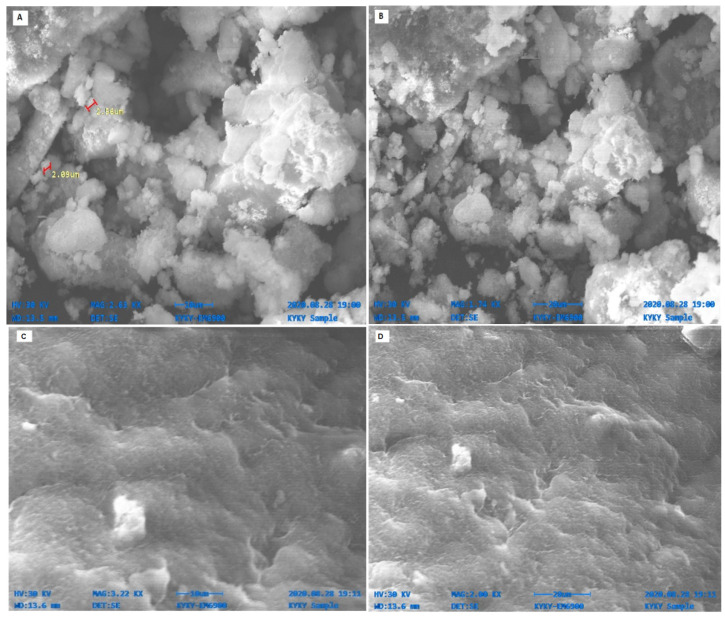
SEM Micrograph of Phytosynthesized ZnO (**A**,**B**) and MgO NPs (**C**,**D**) at different magnification ranges. ZnO NPs were pleomorphic in shape and average size while MgO NPs were ovular and irregularly spherical, assisted by Image J.

Figure 4 shows the SEM micrograph of biosynthesized NPs at different magnification powers respectively. ZnO NPs were pleomorphic in shape and average size was approximately 1 um determined by Image J software. Micro nanoparticles were also observed, which were formed due to the agglomeration of non-stable particles. MgO NPs were ovular and irregularly spherical, while the average size of approximately 1 um was found, assisted by Image J. Some of the nanocrystallite NPs were agglomerated and gave rise to lumps due to the presence of water and other attractive forces.

**Figure 5:**
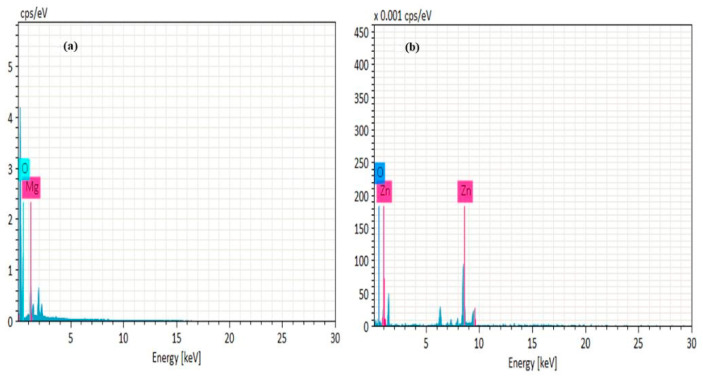
EDX analysis of plant mediated (**a**) MgO and (**b**) ZnO NPs using *D alba*.

EDX analysis was performed to assess elemental composition of ZnO and MgO NPs. Different areas of NPs were focused and corresponding peaks for Mg and O were observed in the sample at 1:1 atomic ratio in range of 0.5 to 1.5 kv indicating the presence of MgO NPs. Furthermore, other signals in the spectrum show elements of biomolecules of extracts acting as reducing and stabilizing agents (Figure 5a). The EDX spectrum (Figure 5b) contains high intensity signals of Zn and O, confirming the successful formation of ZnO NPs, while short peaks were ascribed to the elemental composition of extract biomolecules.

### 2.5. Impact of MgO and ZnO NPs on Learning and Spatial Memory

**Figure 6:**
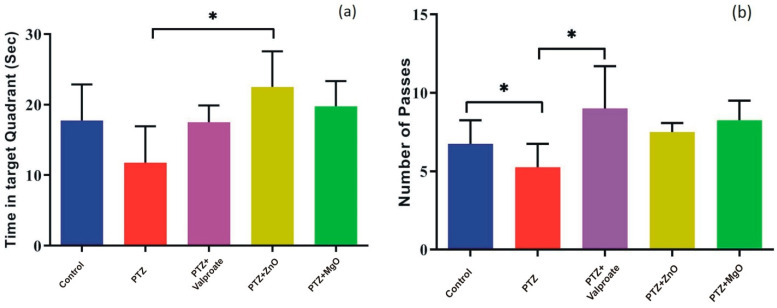
Assessment of spatial learning and memory by MWM test. (**a**) represents time spent by animals in Target quadrant. (**b**) denotes number of passes over target platform. Data represented in Mean ± SEM (*n* = 4 animals in each group), * indicates *p* < 0.05 when PTZ compared to other groups. PTZ; Pentylenetetrazole, ZnO; Zinc Oxide, MgO; Magnesium Oxide.

The analysis of time spent in target quadrant in MWM by one way ANOVA showed the significant interaction between factors “treatment” and “PTZ kindling model” [F (4, 12) = 3.554; *p* < 0.05]. In this regard, a reduction in time spent was observed in PTZ group compared to other groups (*p* < 0.05). In addition, only ZnO group showed significant increase in time spent compared to PTZ group indicating memory boosting effects (PTZ, 11.75 ± 2.594 s; ZnO, 22.50 ± 2.533 s; *p* < 0.05) in post-hoc analysis (Figure 6a). One way ANOVA of the number of passes showed no significant difference between factors [F (4, 15) = 3.027; *p* > 0.05]. However, in the post-hoc test, a significant increase was observed in number of passes in control and valproate treated animals compared to PTZ (PTZ, 5.250 ± 0.7500; Valproate, 9.000 ± 1.354; Control, 6.750 ± 0.7500; *p* < 0.05) (Figure 6b).

### 2.6. Effect of MgO and ZnO NPs on Working Memory

**Figure 7:**
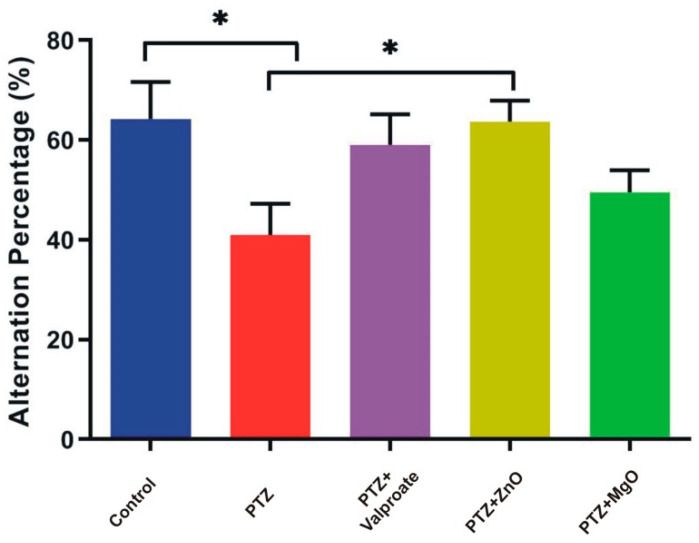
Cognition activity assessment by Spontaneous Alternation in Y Maze Task. All the data expressed in Mean ± SEM (*n* = 4 animals in each group), * *p* < 0.05 when PTZ compared to other groups.

The Y maze test was performed to assess hippocampal dependent spatial working memory. Analysis by one-way ANOVA showed insignificant difference between factors (F (4, 15) = 2.966; *p* > 0.05). However, in post-hoc analysis, we observed significantly high alternation behavior in both the control and ZnO exposed group compared to PTZ, indicating that ZnO treated groups are efficient in holding spatial information for long periods (PTZ, 40.99 ± 6.244; Control, 64.23 ± 7.394; ZnO, 63.67 ± 4.226; *p* < 0.05) (Figure 7).

### 2.7. Effect of ZnO and MgO NPs on Exploratory Behavior and Locomotion

**Figure 8:**
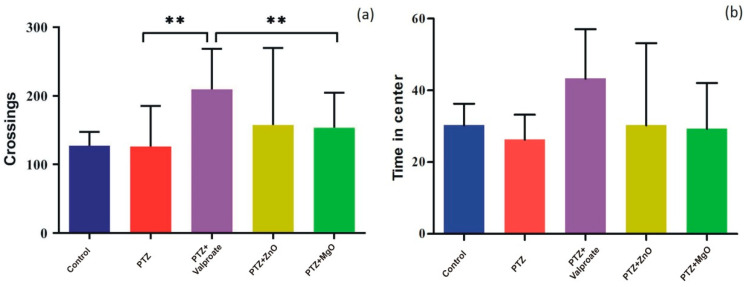

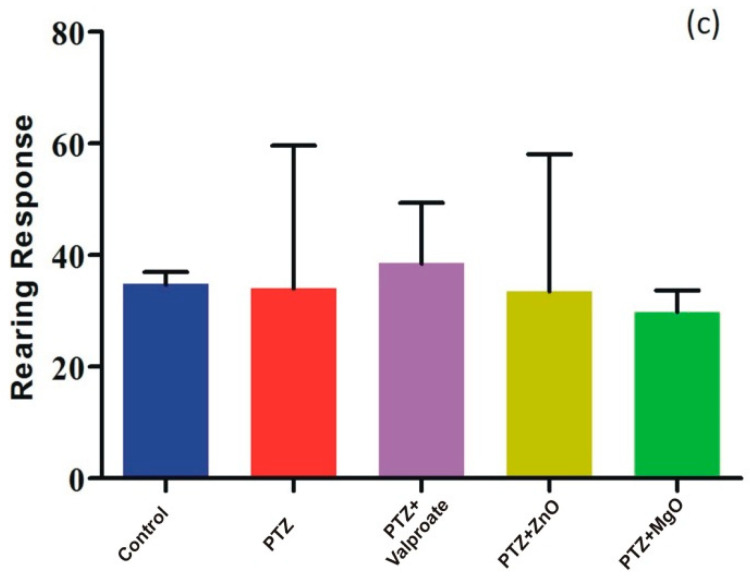
Effects of ZnO and MgO NPs locomotory and exploratory behavior in PTZ- induced kindling model assessed through Open Field test. (**a**) represents crossing of lines in test field, (**b**) denotes time spent by animals in center of the test field, (**c**) depicts rearing response animals. Results expressed in Mean ± SEM (*n* = 4 animals in each group), ** *p* < 0.01 when Valproate group compared to other groups.

In addition to cognition and memory, we evaluated whether ZnO and MgO mitigate PTZ-induced anxiety-like behavior and impaired locomotor activity using OFT. One way ANOVA showed that Treatment with NPs could improve locomotor activity assessed by line crossings though there were no significance [F (4, 15) = 1.003; *p* > 0.05]. However, in the post-hoc test, we observed that only MgO and valproate groups traveled significantly large distance compared to PTZ group (MgO, 153.5 ± 25.62; PTZ, 126.0 ± 29.70; Valproate, 209.3 ± 29.70; *p* < 0.01) (Figure 8a). Similar analysis of the data in Figure 8b,c revealed that ZnO and MgO mitigated anxiety indexes (time in center and rearing response) (F = 0.8408, *p* > 0.05, F = 0.1351, *p* > 0.05), respectively, but failed to achieve statistical significance.

### 2.8. Cognition Improvement Assessments of ZnO and MgO NPs

**Figure 9:**
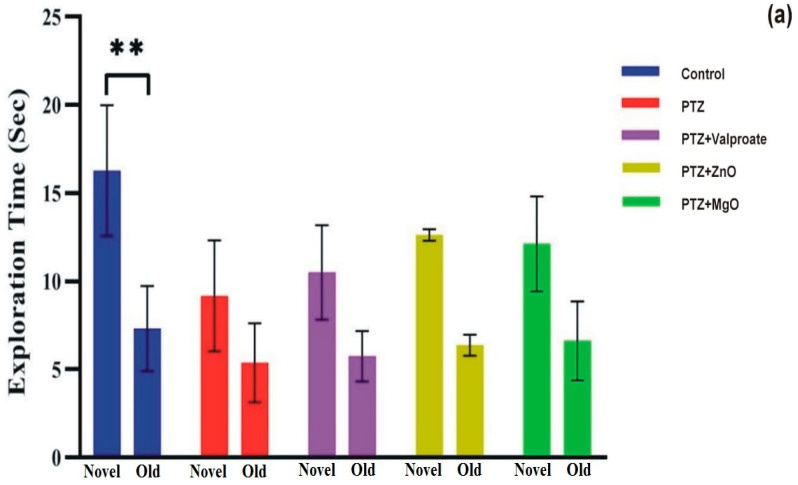

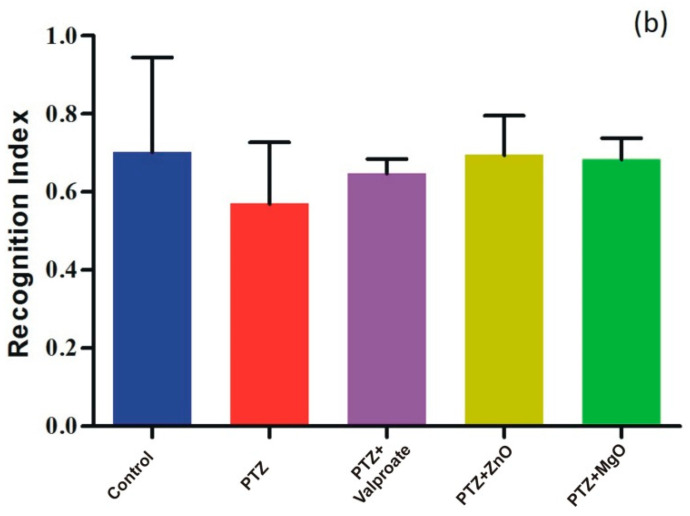
Administration of ZnO and MgO NPs improve Novel object exploration time and RI during NOR test. (**a**) Graphical representation of exploration time, (**b**) depicts Recognition index. Data shown as Mean ± SEM (*n* = 4 animals in each group), ** *p* < 0.01 when Control group compared to other experimental groups.

The cognition enhancement activity of NPs was tested using the NOR test. Figure 9a depicts the two-way ANOVA analysis of exploration time showing significant difference among groups for novel objects (F (1, 15) = 33.43; *p* < 0.0001). Exploration time for novel object was found higher in MgO and ZnO groups confirming its cognition boosting effects. Additionally, post-hoc analysis revealed a significant exploration time of new object compared to others (familiar object, 7.325 ± 2.411; novel object, 16.28 ± 3.707; *p* < 0.01). Furthermore, one way ANOVA analysis of recognition index (RI) followed by post-hoc showed no significant difference between factors (Figure 9b).

### 2.9. Assessment of BBB Integrity

**Figure 10:**
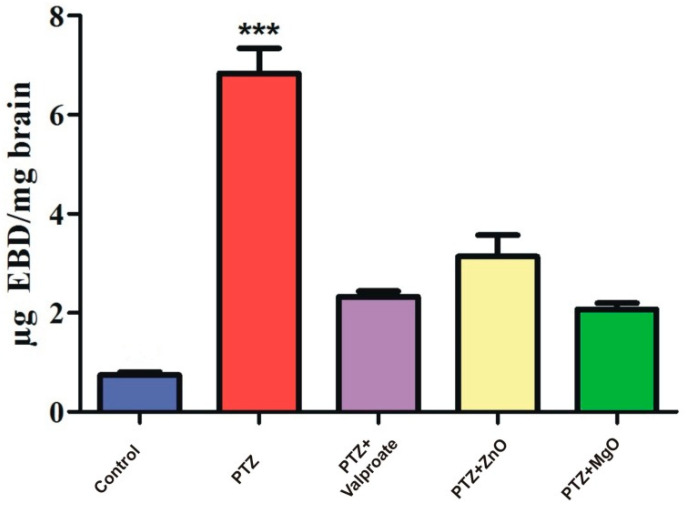
Spectral analysis of tracer (Evan’s blue dye) in Brain of different treatment groups for Blood Brain Barrier leakage. *** *p* < 0.001 when PTZ was compared to other groups.

We also examined the devastating effects of PTZ on BBB vasculature and its recovery through NPs using EBD as a tracer. (Figure 10) represents one way ANOVA analysis followed by post-test which revealed significant difference among the groups [F= (4, 5) = 15.90, *p* < 0.001]. EBD content in PTZ administrated group had significantly high concentration of dye (6.83 ± 0.50 ug EBD/mg, *p* < 0.001) compared to other groups. ZnO and MgO diminished vasculature leakage as dye concentration was found to be lower. Results of the current study clearly indicated that PTZ damages BBB, while ZnO and MgO help in its recovery.

### 2.10. Impact of ZnO and MgO NPs on Body Weight

Table 1 presents the one-way ANOVA of body weight gain, which showed an insignificant difference among the groups (F (2.025, 6.074) = 1.136, *p* > 0.05). However, post-test analysis revealed significant increase in body weight gain of Valproate group compared to other (35.26 ± 0.4787, *p* < 0.001). Furthermore, the mean of initial body weight at the beginning and weight gain at the completion of treatment were significantly different in control and Valproate groups. In the current study, the rate of weight gain in PTZ was slower, while that of the NPs-treated group was faster, indicating negative and positive effects on metabolism, respectively.

## 3. Discussion

Plant mediated biosynthesis is a novel technique for the biogenic synthesis of NPs because of its biodegradability and environmental viability [10]. Nanoparticles have various medical and industrial applications [15,16,17,18,19]. Nano biotechnologists have designed novel agents for delivery of drugs with unique pharmacological effects for neurological disorders. Green ZnO and MgO can be used to regulate metabolism, cytotoxicity, and cellular functions due strong affinity to protein.

In current study, ZnO and MgO were synthesized by a biological method using *Datura alba*, known for having a strong neuroprotective role. The present investigation indicated the post treatment of PTZ-induced kindling with ZnO and MgO NPs to improve memory and BBB integrity damaged by PTZ.

Biomolecules of plants acting as reducing and capping agents were reported in previous literature. The optical properties of metal nanoparticles depend on shape, size, composition, and interaction between the particles and surfactants. The successful formation of NPs was confirmed by different characterization techniques. UV-vis spectroscopy of biosynthesized ZnO and MgO showed characteristic peaks at 380 nm and 242 nm respectively which supported by [20,21]. Our result clearly showed the reductive role of biomolecules in ZnO and MgO NPs formation. FTIR analysis was carried out for the determination of functional groups present on ZnO and MgO NPs surface acting as capping agents. It revealed the presence of carboxylic acid, ether, ester, phenol, flavonoids, aldehyde, ketones, protein, and enzymes in plant extracts which reduced metal ions and stabilized NPs which was concurred with the observation of [10,22,23]. XRD was performed for the determination of crystalline structure of NPs. The planes compared with (JCPDS) ZnO pattern (file no: 043-0002) revealed hexagonal Wurtzite structure of ZnO NPs [24]. obtained similar crystalline structure of phytoassisted ZnO NPs. Crystalline nature of MgO NPs was confirmed by matching planes with JCPDS data card no: 002-1207. Partial amorphous state and presence of water with impurities were also observed which are indicated by some unexpected peaks [25]. observed similar planes for plant mediated MgO NPs. XRD analysis of the current study reveals that biometabolites of plant are responsible for maintaining crystalline structure and avoid agglomeration of particles. SEM analysis showed average particle size of phytoassisted ZnO and MgO NPs was 1um with pleomorphic and oval shapes respectively [26]. have reported biosynthesized MgO NPs with similar size and shape. The study of [17,27] revealed that using zinc acetate as precursor for biogenic ZnO give rise to large flower shaped NPs which is in close agreement with the current work. The agglomeration of NPs took place either due to high surface energy, instability, or attractive forces caused by the concentration of extracts, pH, and temperature. EDX profiling of current NPs concurred with [28,29] for plant mediated MgO and ZnO NPs respectively.

Neuroprotective role of ZnO and MgO regarding memory, learning and anxiety have been studied in various models. It was reported that Magnesium and Zinc are neuromodulator of glutamate and NMDA receptor signaling pathways which are responsible for learning, memory, anxiety, and seizure. In the CA1 region, Magnesium activates NMDA receptors, leading to an increase in synaptic plasticity, inducing LTP, as well as improved learning and memory [30]. However, in the current study, we provided new information regarding the impact of ZnO and MgO NPs in respect of cognitive deficits and behavioral improvement in a PTZ model. The MWM, Y Maze, and NOR test were adopted to evaluate memory and learning capabilities. In the MWM test, we found increase in number of passes and time spent in target quadrant in ZnO and MgO NPs groups indicating memory improvement compared to PTZ group. Previously, a similar increase was observed in depression- and Atropine-induced memory impairment mice models [31,32]. The role of zinc in memory formation through NMDA and GABA receptors is indicated by different in vivo studies. High intracellular zinc concentration and its entry to postsynaptic neuron improve LTP in mossy fibers CA3 hippocampus and synaptic plasticity which are governing mechanisms for memory and learning [33]. Data analysis of the Y Maze and NOR tests showed that the exposure of epileptic mice to ZnO and MgO increases the percent alternation behavior and exploration of novel objects, suggesting a memory boosting effect of NPs. The previous observation of [34,35,36,37,38] was found in line of current results.

An accumulation of zinc in synaptic vesicles and its release to postsynaptic neuron from glutamatergic neuron modulate GABA and NMDA receptors. Both receptors are also considered as important factors in the formation of anxious behaviors. Hyperactivation of NMDA caused by the excessive secretion of glutamate induces anxiety, which could be ameliorated by the administration of zinc. Moreover, regarding anxiety, the inhibition of presynaptic glutamate release can be achieved through the intensification of GABA secretion by zinc in the hippocampus [35]. In OFT, we observed an increase in the number of line crossing, time in center, and rearing response in ZnO and MgO treated groups. Our findings were in close agreement with results reported by [32,36,37,38,39], as they observed significant improvement in drug induced anxiety model.

Therefore, it could be speculated that the administration of ZnO and MgO NPs possibly modulated the above mechanisms and thus enhanced memory, learning, and mitigated anxiety. Epileptic seizures and pathology disrupt the BBB, allowing neurotransmitters, polar compounds, and proteins to directly affect the brain [37,38,39,40,41,42]. In the present research work, EBD was injected to assess BBB permeability, which was found in higher concentration in kindled, showing the devastating effects of the drug. The BBB protecting effects of biogenic ZnO and MgO NPs remain unavailable. However, our data show that phytosynthesized MgO and ZnO NPs have good healing effects on the BBB.

## 4. Materials and Methods

### 4.1. Drugs and Chemicals

Pentylenetetrazole was purchased from Alfa Aeser (Tianjin, China) while Valproic acid (tablets), methanol, and ethanol were obtained from the local market. Paraformaldehyde, Evan’s Blue dye, Sodium hydroxide, Zinc acetate, Sucrose, and Magnesium nitrate were procured from Sigma-Aldrich (St. Louis, MI, USA).

### 4.2. Extract Preparation

Prior to the collection of *Datura alba* Leaves from different wild regions of KPK, Pakistan. Permission was ascertained from the Department of Zoology Abdul Wali Khan University Mardan and Forestry Department of KPK so that the study could comply with relevant institutional, national, and international guidelines and legislation.

The wild leaves of *D. alba* were identified and authenticated by Dr. Mohib Shah in the Department of Botany, Faculty of Chemical and life Science, Abdul Wali Khan University Mardan Kp Pakistan. The leaves of *D. alba* specimen were deposited in the institutional herbarium with voucher number AWKUM. BOT.54.3.14.

**Figure 11:**
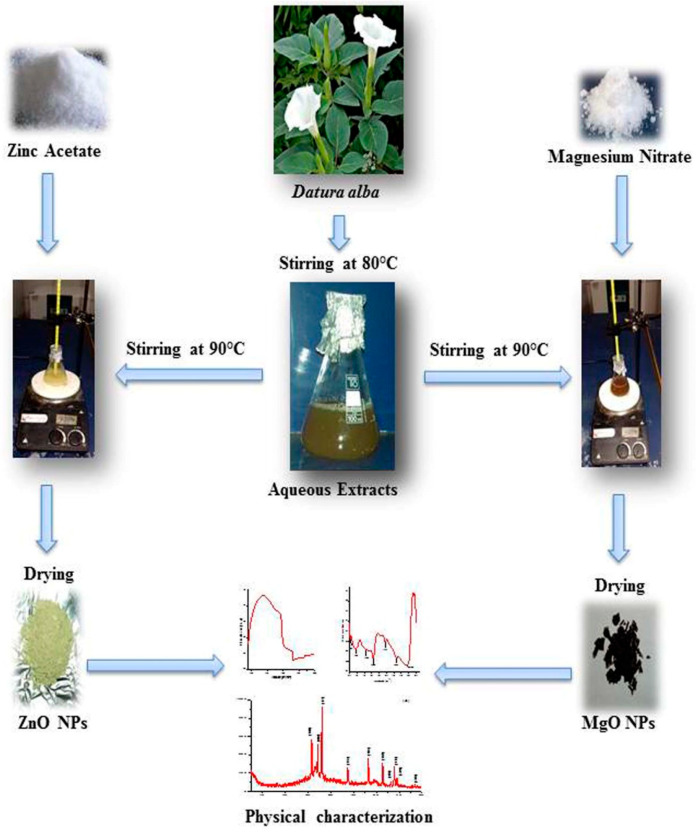
Scheme synthesis of MgO and ZnO NPs using *Datura alba* leaves Extracts.

Leaves of *D. alba* were washed with distilled water thoroughly, dried and pulverized into fine powder. Powder weighing 5 g was boiled in 100 mL distilled water in 500 mL sealed conical flask at 80 °C for 50 min hour on Hotplate. The obtained solution was cooled at room temperature, filtered, and the aqueous filtrate was stored at 5 °C. The scheme of NPs synthesis is shown in (Figure 11).

### 4.3. MgO NPs Synthesis

For the biosynthesis of MgO NPs, procedure of [27] was adopted with minor modifications. We added 250 mg of Mg(NO_3_)_2_ salts to 5 mL of dH_2_O. Then, 1:1 solution and leaf extracts were mixed in a 50 mL flask. The solution was stirred for 4 h at 90 °C using a magnetic hotplate. The mixture’s color changed, and the resulting NPs were separated by centrifugation at a speed of 10,000× *g* for 15 min. The pellet was washed with ethanol and dH_2_O many times to remove impurities. Furthermore, the NPs were dried overnight at 80 °C in an oven and stored for analysis.

### 4.4. ZnO NPs Synthesis

ZnO NPs were biosynthesized by slightly modifying the method of [25]. A 1:1 ratio of 0.27 M Zinc acetate solution and leaves extract was mixed in a flask. The pH of the mixture was maintained at 12 by pouring 2 M NaOH and subjected to stirring on magnetic hotplate at 85 °C for 2 h until color changed. The NPs suspension was centrifuged for 10 min at 14,000× *g* to collect NPs and remove extra extract. NPs were purified from unwanted residues with dH_2_O and ethanol, followed by overnight drying at 80 °C and preservation for further examination.

### 4.5. Biosynthesized NPs Characterization

The formation of both biogenic NPs was confirmed and characterized by UV-vis spectroscopy (spectrophotometer Cary 500, SpectraLab, Toronto, Canada). Fourier transformed infrared spectroscopy (FTIR) (Nicolet 380; ThermoFisher Scientific, Seoul, Republic of Korea) was used to determine various functional groups in NPs. Crystalline nature was assessed by X-ray diffractometry using (Difray, Saint Petersburg, Russia). SEM microscopy coupled with an EDX facility was used for morphology and elemental composition analysis.

### 4.6. Animals and Condition

In current study, healthy, adult BALB/C male mice (25–30 g, 5 weeks old, *n* = 20) were provided by the National Institute of Health, Islamabad. The animals were housed under controlled standard condition (22 °C ± 2 °C, 60% relative humidity, 12 h Dark/light period) and given access to standard food and water *ad libitum*. The experimental protocol was approved by the Research grants and Experimentation Ethics Committee of the Department of Zoology Abdul Wali Khan University Mardan on the use of Animal samples. Moreover, it was carried out in strict compliance with the National Research council guidelines on the care and use of laboratory.

Each procedure followed the rules established by the Institutional Ethical Committee of Abdul Wali Khan University Mardan, Pakistan for the handling and use of animal sample.

### 4.7. PTZ Kindling Model: In Vivo Epileptogenesis

Kindling model was developed by intraperitoneal injection of PTZ (40 mg/kg; sub-convulsive dose) on every second day for 15 days and seizure intensity were monitored for 35 min after injection. The stages of seizure intensity were assessed by using Racine’s scale scoring as follows: 0; “normal behavior” 1; “lying on belly, immobilization” 2; “limbs myoclonus, head nodding” 3; “holding tail up, myoclonus of whole body” 4; “falling down, tonic seizure and rearing” 5; “jumping, rushing, tonic-clonic seizure” 6; “Death”. Animals were considered kindled showing stage 5 or 6 on three consecutive PTZ administrations [20].

### 4.8. Study Design

**Figure 12:**
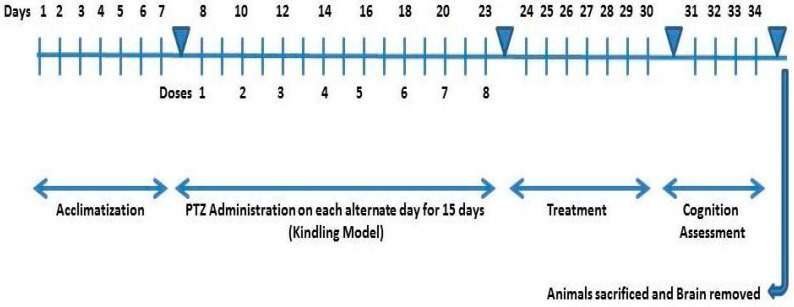
Schematic representation of experimental design.

The animals were divided into five study groups randomly (*n* = 4). Four groups were exposed to PTZ to develop a successful kindled model. Each group was post-treated with NPs and standard drug for one week as follows; Group 1: Intact, Saline throughout experiment, Group 2: PTZ group, received saline, i.p, Group 3: Valproate group, administrated with 200 mg/kg, i.p., Group 4–5: ZnO and MgO NPs groups, received 10 mg/kg, i.p. respectively. (Figure 12) depicts a schematic representation of the experimental procedure.

### 4.9. Neurobehavioral Studies

#### 4.9.1. Morris Water Maze Task

The MWM task was conducted to assess spatial learning and memory as demonstrated by [21]. Animals were trained with visible and hidden platform in a round blue pool (diameter: 70 cm, depth: 30 cm) filled with warm water (maintained around 26 ± 2 °C) and divided into four equal quadrants. An escape platform (diameter: 12 cm, height: 20 cm) was placed in middle of one quadrant. Different proximal and distal cues were placed around the pool to facilitate navigation. Three trials per day for 60 s were given from different quadrants and behavior was video recorded by camera installed above the maze. Platform was visible on day 1st while hidden for 2nd and 3rd day of training phase. Failed animals were guided manually to find platform after 60 s. A probe trial was conducted after treatment where water was made opaque by adding skim milk. The number of passes through the platform quadrant and time spent was recorded.

#### 4.9.2. Y-Maze Test

To evaluate working memory, a Y-maze spontaneous alternation behavior test was conducted as described by [22]. Y maze is composed of three arms equally spaced (angle: 120°) and were labeled as A, B, and C. A five-minute trial was given to each animal for arm exploration, and the number of alternations and entries were manually noted. Successive entry by an animal to all the arms is termed as Alternation. The following formula was used to calculate percent alternation rate:

Percentage Alternation = spontaneous alternations/(Number of arms entries 2) × 100

#### 4.9.3. Open Field Test

The OFT was carried out for assessing locomotor activity, anxiety, and exploration by following the method of [23]. The open field is simple apparatus made of Plexiglas box which is 70 cm × 70 cm with 70 cm height. Black lines on box floor were drawn with marker to divide the field into 16 equal squares. Peripheral and central squares were black and white respectively. Each animal was placed in the central area and the following behaviors were observed: time in central area, locomotion (line crossing) and rearing; (standing on hind legs).

#### 4.9.4. Novel Object Recognition Test

The NOR task was conducted to assess short term memory and cognition improvement of mice as demonstrated by [4]. The apparatus is a square plastic box (55 cm × 55 cm) with walls 55 cm in height. The testing session consisted of T1 and T2 trials, each with a 3-min duration. In T1, animals explored two similar objects while T2 was started after 4 h where one object was replaced with novel. Interaction time with objects in both trials was noted and recognition index was calculated using formula RI = *T_novel_/T_novel_* + *T_old_*. The greater the RI value of an animal, the better the short-term memory.

#### 4.9.5. Evan’s Blue Dye (EBD) Leakage Assay

Evans blue dye is an inert tracer used to assess leakage of the BBB. Leakage was assessed by injecting 2% EBD solution (200 μL/animal) in caudal vain and its circulation allowed for 35 min. Then, animals were perfused with 40 mL PBS for 15 min under Diethyl ether Anesthesia. The brain was isolated, weight measured and 1200 μL of PBS was added for homogenization. The homogenized brain was sonicated followed by centrifugation at 4 °C for 35 min at 14,000 rpm. Afterwards, 500 μL of 50% Trichloroacetic acid was poured to equal amount of supernatant for protein precipitation following by overnight incubation at 4 °C. On next day, centrifugation was performed again at same temperature for same time at 14,000 rpm. Dye quantity was analyzed by absorbance at 610 nm with the help of spectrophotometer. Standard curve was used to express EBD in μg/mL [24].

### 4.10. Statistical Analysis

GraphPad Prism (Version 8) Software 2365, San Diego, CA, USA, was used for the statistical analysis of numerical data. Data were expressed as Mean ± SEM. The ANOVA (one- or two-way) test was performed to evaluate statistical significance, followed by Bonferroni’s post-test for multi group comparisons. The level of significance was set at *p* < 0.01, *p* < 0.05 and *p* < 0.001.

## 5. Conclusions

In summary, this study demonstrated that ZnO and MgO NPs could be generated successfully by using primary and secondary biometabolites of *D. alba.* Phytochemical constituents of leaves not only support the reduction and stabilization of NPs, but also showed synergistic effects against cognition improvement due to their bioactive components. These NPs can be synthesized from *D. alba* leaf extracts and used as therapeutic agents. They could be readily used for the treatment of neurological as well as metabolic disorders and pathogenicities. Further studies are needed to identify bioactive biomolecules, determine the underlying action mechanism of NPs, and to develop a potential regimen to attenuate epilepsy-associated comorbidities.

## Figures and Tables

**Table 1:** Comparison of gained and initial body weight of animals injected with various doses of Valproate, PTZ, MgO and ZnO NPs.

Treatment Groups	Dose mg/kg	Initial Weight (g)	Gained Weight (g)
PTZ	40	31.01 ± 0.4079	34.31 ± 1.610
Control	Saline	28.80 ± 1.110	35.01 ± 1.230 **
Valproate	200	31.01 ± 0.4078	35.26 ± 0.4787 ***
MgO	10	32.01 ± 1.236	36.80 ± 0.9470
ZnO	10	22.51 ± 6.560	33.26 ± 1.501

The results are expressed in Mean ± SEM (*n* = 4 animal/group). One way ANOVA was performed for statistical analysis of data. PTZ; Pentylenetetrazole, MgO; Magnesium Oxide, ZnO; Zinc oxide. ** indicates *p* < 0.01 and *** *p* < 0.001.

## Data Availability

The data presented in this study are available on request from the corresponding author.

## Sample Availability

Samples of the compounds are not available from the authors.
